# Nutritional and Functional Evaluation of *Inula crithmoides* and *Mesembryanthemum nodiflorum* Grown in Different Salinities for Human Consumption

**DOI:** 10.3390/molecules26154543

**Published:** 2021-07-27

**Authors:** Alexandre R. Lima, Florinda Gama, Viana Castañeda-Loaiza, Camila Costa, Lisa M. Schüler, Tamára Santos, Miguel Salazar, Carla Nunes, Rui M. S. Cruz, João Varela, Luísa Barreira

**Affiliations:** 1 MED-Mediterranean Institute for Agriculture, Environment and Development, Universidade do Algarve, Campus da Penha, 8005-139 Faro, Portugal; carlima@ualg.pt (A.R.L.); fmgama@ualg.pt (F.G.); rcruz@ualg.pt (R.M.S.C.); 2 CCMAR-Centre of Marine Sciences, Campus de Gambelas, Universidade do Algarve, 8005-139 Faro, Portugal; vcloaiza@ualg.pt (V.C.-L.); cqcosta@ualg.pt (C.C.); lmschueler@ualg.pt (L.M.S.); tfsantos@ualg.pt (T.S.); jvarela@ualg.pt (J.V.); 3 RiaFresh, Sítio do Besouro, CX 547-B, 8005-241 Faro, Portugal; miguel.salazar@agro-on.pt (M.S.); carla.nunes@agro-on.pt (C.N.)

**Keywords:** halophytes, soilless cultivation, salinity stress, nutritional composition, bioactive compounds, sensory profile

## Abstract

The nutritional composition and productivity of halophytes is strongly related to the biotic/abiotic stress to which these extremophile salt tolerant plants are subjected during their cultivation cycle. In this study, two commercial halophyte species (*Inula crithmoides* and *Mesembryanthemum nodiflorum*) were cultivated at six levels of salinity using a soilless cultivation system. In this way, it was possible to understand the response mechanisms of these halophytes to salt stress. The relative productivity decreased from the salinities of 110 and 200 mmol L^−1^ upwards for *I. crithmoides* and *M.* *nodiflorum*, respectively. Nonetheless, the nutritional profile for human consumption remained balanced. In general, *I. crithmoides* vitamin (B1 and B6) contents were significantly higher than those of *M. nodiflorum*. For both species, *β*-carotene and lutein were induced by salinity, possibly as a response to oxidative stress. Phenolic compounds were more abundant in plants cultivated at lower salinities, while the antioxidant activity increased as a response to salt stress. Sensory characteristics were evaluated by a panel of culinary chefs showing a preference for plants grown at the salt concentration of 350 mmol L^−1^. In summary, salinity stress was effective in boosting important nutritional components in these species, and the soilless system promotes the sustainable and safe production of halophyte plants for human consumption.

## 1. Introduction

Halophytes are plants known to grow in naturally saline habitats, where 99% of salt-sensitive species die due to NaCl toxicity [1]. Furthermore, despite the interspecific variability found, it is known that the biomass of several halophyte species have shown good phenolic contents, and an ability to scavenge superoxide anions or other free radicals [2,3]. As a result, these plants have become excellent candidates for human consumption, with properties suitable for pharmacological use, boosting the interest of producers and consumers in the cultivation of these species.

*Inula crithmoides* L. (syn. *Limbarda crithmoides* L. Dumort.) is a halophyte belonging to the family Asteraceae. It is a dicotyledonous succulent plant commonly found in saltwater marsh areas on the Mediterranean basin [4]. *Mesembryanthemum nodiflorum* L. is another halophyte that grows in a similar habitat and is a species belonging to the Aizoaceae family, which has widespread use in traditional Tunisian medicine as an antiseptic for oral infections, stomach problems, wounds, and burns [5]. In Portugal, both species are found along the coastal area and in saltwater marshes, which are zones with extremely saline soil. The tolerance of these species to salt is associated with their osmoregulatory function from the accumulation of betaine, glycine, and proline [6]. In addition, both plants have large contents of secondary metabolites exhibiting different bioactivities, such as those shown by *Inula* spp. extracts with proven antibacterial, antioxidant, antifungal, and cytotoxic activities [7], while *Mesembryanthemum* spp. extracts are associated with antioxidant and antiviral activities [3,8].

In a more general context, both wild halophyte plants and those cultivated under controlled conditions have a nutritional profile suitable for human consumption, with low fat content and high concentrations of protein, fiber, and minerals [9], being rich in bioactive compounds [10] as well. In this sense, the growing interest in halophytes for human consumption and pharmacological and cosmetic use not only places these plants in a prominent position at the scientific level but also in an industrial context. However, there is a justifiable concern that increased knowledge of the health benefits of halophytes may lead to their abusive collection from natural habitats, causing an environmental imbalance.

Hence, cultivation using salinized soils not suitable for conventional agriculture or cultivation in soilless systems as a sustainable and eco-friendly solution presents itself as a crucial strategy to not only preserve the environment from possible consumers of these plants but also to promote the agricultural production sector. This type of agriculture is particularly interesting in areas characterized by limited availability of water [11], as is the case of the Algarve, a region in the south of Portugal with growing interest in the production of halophyte plants. Cultivation in soilless systems has additional advantages in terms of increased productivity, product uniformity, and food security [11,12], as it enables the control and avoidance of hyperaccumulation of heavy metals by several halophyte species [13]. Currently, in Portugal, there are several ventures for halophyte production using different cultivation methods, which include controlled soil, hydroponics, and soilless systems. One of the most successful ventures is RiaFresh, which produces *Inula crithmoides*, *Mesembryanthemum nodiflorum*, and *Salicornia ramosissima* J. Woods, among other species, all cultivated using a soilless system and aimed for human consumption.

Since in halophyte plants, the accumulation and synthesis of nutrients are generally stimulated in response to biotic and abiotic stresses such as salinity [14,15], this study aimed to evaluate the influence of salinity on productivity and the nutritional and sensory properties of *I. crithmoides* and *M. nodiflorum* grown in greenhouses using a soilless system. This information was used to find the conditions for the optimal cultivation of *I. crithmoides* and *M. nodiflorum*, not only in terms of productivity, but also to obtain a balanced nutritional composition, attractive sensory characteristics, and a suitable content of bioactive compounds. Both species are commercially produced and used in gourmet cuisine; the growing interest of consumers on these plants and their relevance for the Mediterranean diet as sustainable sources of fiber, proteins, together with the poor knowledge regarding the most appropriate cultivation conditions for the optimum nutritional properties motivated this study. Taken together, the results here reported will contribute to a sustainable culture while promoting the consumption of these plants as a healthier substitute for salt.

## 2. Results and Discussion

### 2.1. Effects of Salinity on Productivity

**Figure 1:**
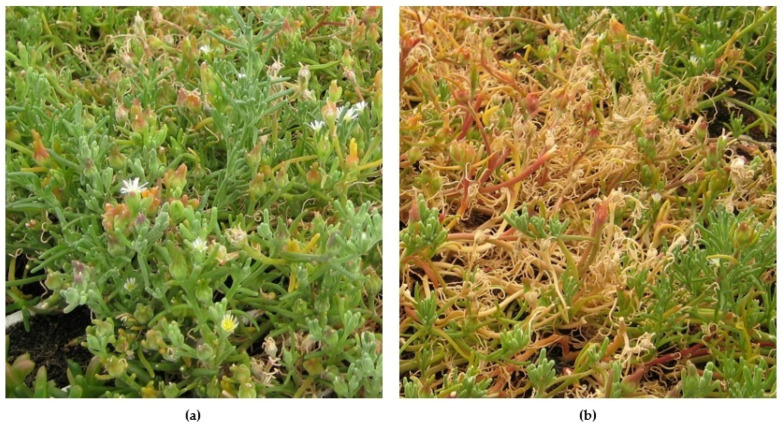
*M. nodiflorum* cultivated with the 35 mmol L^−1^ (**a**) and 465 mmol L^−1^ (**b**) nutrient solutions. The accumulation of reddish pigments in the stems becomes evident as the salinity is increased.

Upon testing different growth conditions, plant growth was mainly affected by salinity. I. crithmoides showed a significant decrease in plant height for salinity levels over 35 mmol L^−1^. Conversely, *M. nodiflorum* plants were more comparable in size at different salinities, although a reddish color in the stems (Figure 1) could be observed, becoming more intense at higher salinities. This is a trait often observed in plants of this genus and is generally associated with the accumulation of water-soluble pigments belonging to the betacyanins group, which are commonly produced in response to salinity [16]. Moreover, the response to salinity was species-dependent, as salinity clearly compromised relative productivity to a greater extent in *I. crithmoides* as compared to what was observed in *M. nodiflorum* (Table 1).

In this study, *I. crithmoides* had maximum productivity at the lowest salinity (35 mmol L^−1^), whereas for *M. nodiflorum*, the maximum productivity was attained between salinities of 35–110 mmol L^−1^. In *I. crithmoides*, the relative productivity decreased by 57% when the salinity was increased from 35 to 110 mmol L^−1^. Regarding the highest salinity (465 mmol L^−1^), the relative productivity decreased by 66% and 42%, respectively, for each species. These results show a stronger impact of salinity on productivity than the findings of Zurayk and Baalbaki [4], where the growth of potted *I. crithmoides* at different levels of salinity for a period of 87 days was only affected by water with an electric conductivity (EC) exceeding 20 dS m^−1^ (salinity ≈ 200 mmol L^−1^). Although, in a study on *Inula viscosa* L. by Curadi et al. [17], they observed a consistent decrease in biomass production caused by salt; irrigation water with an EC of 12 dS m^−1^ (salinity ≈ 120 mmol L^−1^) led to a 42% decrease compared to control plants where salt addition was omitted. Regarding *M. nodiflorum*, we were not able to find a comparable study, but with a related species, *M. crystallinum*, Atzori et al. [18] observed that seawater irrigation up to an EC of 35 dS m^−1^ did not impact plant growth. Although *M. nodiflorum* does not have enlarged unicellular trichomes in the leaves and stems, also called bladder cells, as *M. crystallinum*, it has narrow succulent-like leaves that also act as water reservoirs, providing protection from short-term high salinity or water deficit stress.

### 2.2. Proximate Composition and Firmness

The effect of salinity on the proximal composition of *I. crithmoides* and *M. nodiflorum* is shown in Table 1. The moisture content of both plants was similar at low (35 mmol L^−1^) and at high (465 mmol L^−1^) salinities. However, the increase in the amount of salt available to the plants promoted a significant reduction in the moisture content of both species. These results corroborate those obtained in similar studies with other halophyte species, such as *Salicornia ramosissima*, *S. europaea* and *S. persica.* Indeed, this lower moisture content can be attributed to the osmotic imbalance caused by the reduction in water uptake by the plant cells upon exposure to large amounts of NaCl [19,20].

The salt concentration of the cultivation medium significantly influenced ash content. According to Attia-Ismail [21], a high ash content is typical of halophytes, and in this case, can be attributed to the large capacity for mineral accumulation by these plants. In this study, *I. crithmoides* and *M. nodiflorum* had the highest ash content, 5.70 and 5.04 g 100^−1^ wet weight (ww), respectively, at 465 mmol L^−1^ (Table 1). However, in addition to the significant difference observed between species, with *I. crithmoides* presenting the highest content, *M. nodiflorum* ash content remained approximately constant up to the salinity of 350 mmol L^−1^, suggesting that this species is able to counteract the accumulation of salts in its tissues until the latter salinity is reached. Conversely, *I. crithmoides* ashes underwent a gradual increase, strongly suggesting that this species may use a different regulatory mechanism for osmoregulation.

*Inula crithmoides* had higher amounts of protein than *M. nodiflorum*. Moreover, an increase in the crude protein content, around 14–19% compared to the samples cultivated at the lowest salinity, was observed in plants cultivated at the three highest salinities tested (275, 350, and 465 mmol L^−1^), indicating that *I. crithmoides* was significantly affected by the increase in salt stress (Table 1). A similar response was reported by Agudelo et al. [13] for three species of halophytes (*Atriplex halimus*, *Salicornia fruticosa*, and *Cakile maritima*) under salt stress between 0 and 300 mmol L^−1^. Salinity appears to have had a lower impact on the protein content of *M. nodiflorum*, as no significant differences between samples were observed. Similarly, Lima et al. [20] and Ventura et al. [22] observed that the protein contents of *S. persica* and *S. ramosissima* were also unaffected by salt stress. Although these results suggest an interesting stability in terms of total protein content, many studies have reported that halophytes can display distinct coordinate proteomic responses to salinity. Indeed, besides their role in the structural organization of the cell and metabolism, proteins are key regulators of cellular responses to salt stress, which may range from the induction of heat shock proteins with the ability to avoid protein denaturation to higher expression of genes encoding ion transporters [23,24,25].

Overall, the lipid content of these halophyte plants was low [26]. However, there is a trend toward an increase in lipids in plants grown under greenhouse and outdoor farming conditions compared to wild plants [9,27]. In this study, despite the range of salt stress levels used, the lipid contents of both species grown at different salinities were not strikingly different. Only the plants cultivated at 110 mmol L^−1^ had higher fat contents than those at other salinities (Table 1). Total dietary fiber (TDF) includes, in addition to oligosaccharides and polysaccharides, other classes of associated plant molecules in their composition, such as cutins, lignins, and suberines [28,29]. Several studies have reported that wild halophyte plants tend to have higher fiber contents than plants grown under controlled conditions [9,20,30]. One factor that stimulates the production of structural fibers in wild halophytes is exposure to abiotic conditions, especially wind, which is often absent in controlled environments such as greenhouses. Conversely, controlled cultivation conditions provide a greater supply of nutrients, increasing the growth rate and reducing the investment in the production of structural polysaccharides, such as cellulose and lignin [19,20]. In this study, both species cultivated at 275 mmol L^−1^ showed higher fiber content. However, under higher salt stress (350 and 465 mmol L^−1^), the plants showed a slight decrease in fiber content, hinting that mild salt stress can stimulate the production of structural components by plants, but excess salt reduces the production of structural polysaccharides.

The firmness parameter for *I. crithmoides* ranged from 181 ± 19 (35 mmol L^−1^) to 154 ± 10 (465 mmol L^−1^) and for *M. nodiflorum* from 258 ± 16 (35 mmol L^−1^) to 186 ± 32 (465 mmol L^−1^), as shown in Table 1. In sensory and gastronomic terms, firmness is one of the main physical properties that explain the texture of a product [30]. For the studied halophytes, this property was used to evaluate the substantial resistance to deformation and hardness of the plants and by how much salinity can influence these characteristics. Overall, firmness was inversely correlated with cultivation salinity, again a strong indication that salt stress conditions reduce the formation of structural components of plants (i.e., cellulose and lignin), decreasing their firmness and influencing their palatability [9,19]. This result is unlike what is usually seen in edible fibers, which do not include cellulose, but include other small oligosaccharides that may have been induced in response to exposure to moderate concentrations of salt. Moreover, the significantly higher firmness of *M. nodiflorum* plants compared to that of *I. crithmoides* can be attributed to other characteristics such as the presence of a stomatal complex capable of performing day and night movements for osmotic regulation, water absorption, and accumulation of carbon dioxide [30], different from those present in the lanceolate and cracked leaves of the *I. crithmoides* [4].

### 2.3. Minerals

The minerals composition of the studied halophyte species is shown in Table 2. As expected, Na was the element present in larger quantities in both halophyte species. K was the second most abundant element in this analysis. In both plants, Na content was approximately twice as high in the condition of maximum salt stress (465 mmol L^−1^) compared to that of plants cultivated at the lowest salinity (35 mmol L^−1^). This increase was not seen for the K, Ca, and Mg.

The increase in Na and the reduction in K, Ca, and Mg is reported in the literature for other species of halophyte plants subjected to different levels of salt stress during cultivation [13,20]. According to Flowers and Colmer [1], the decrease in K, Ca, and Mg can be associated with an inhibition of the accumulation of other macroelements due to excess sodium, resulting in an increase in the Na:K ratio upon exposure to salt stress, as observed in the studied species (Table 2). A similar link was observed by Katschnig et al. [31] in *Salicornia dolichostachya*. Other studies have observed that NaCl stress promotes a competition between Na+ and K+ upon their uptake by the plant, being responsible for influencing the activation of osmolytes that seek the osmotic balance of the plant, promoting a drop in the endogenous K+ levels [32,33].

The content of Fe in *I. crithmoides* increased from 8.78 to 12.3 µg g^−1^ in plants grown at the highest salinity (465 mmol L^−1^). From a nutritional point of view, this sample presented approximately 1.2 mg 100 g^−1^ of nonheme iron (commonly present in plant-based foods). This result reveals that this halophyte may be a good source of Fe, when compared to the 2.35 mg 100 g^−1^ present in spinach, a vegetable commonly seen as a good Fe source [34]. *M. nodiflorum* proved to be a suitable source of Mn, accumulating this mineral as saline stress increased, doubling the content of this element, from 5.31 to 10.1 µg g^−1^ when comparing plants cultivated at 35 and 465 mmol L^−1^, respectively. The presence of these and other essential minerals adds nutritional value to these plants as these are indispensable for the maintenance of certain biochemical and physiological processes in the human body [24,32]. *M. nodiflorum* grown at salinities of 200, 275, and 465 mmol L^−1^ accumulated Zn at concentrations of 19.4, 58.4, 43.0 µg g^−1^, respectively. These values were significantly higher than those found for *I. crithmoides*, in the present study, or for other halophytes subjected to salt stress, such as *Atriplex halimus*, *Salicornia fruticosa*, *S. ramosissima,* and *Cakile maritime* [13,20]. This accumulation capacity of Zn has been reported for several plant species of the families *Brassicaceae*, *Caryophyllaceae*, and *Dichapetalaceae* [35]. Furthermore, it is known that greenhouse cultivation favors high mineral absorption [36], and in this case, the combination of greenhouse cultivation and the natural ability of the species to uptake this element can influence Zn accumulation.

From a food security perspective, the concentrations of toxic metals were generally low, with Pb for *M. nodiflorum* showing concentrations below the limit established by legislation, 0.30 µg g^−1^ [37], and Cd was not detected in either species (LOD: 0.02 µg L^−1^).

### 2.4. Vitamins and Carotenoids Content

**Figure 2:**
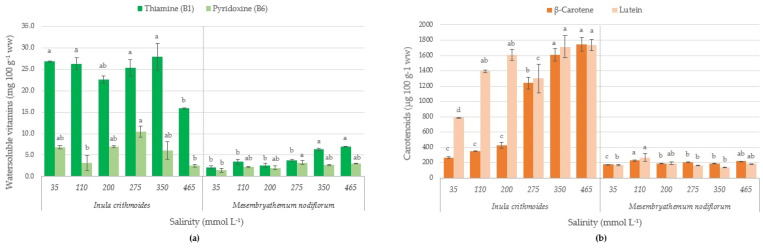
(**a**) Vitamin content and (**b**) carotenoid content of *I. crithmoides* and *M. nodiflorum* cultivated at six different salinities. LOD: B1-2.6 µg mL^−1^, B6-2.1 µg mL^−1^, *β*-Carotene-8.2 µg mL^−1^, Lutein-1.4 µg mL^−1^. In each chart, bars of the same color and labeled with different letters are significantly different (*p* < 0.05).

Vitamin and carotenoid contents of the studied plants are shown in Figure 2. In general, vitamin and carotenoid levels were species-dependent as *I. crithmoides* presented almost three and four times more vitamin B1 and lutein, respectively, regardless of the salinity, when compared with those of *M. nodiflorum*. Concerning *β*-carotene, the content of this carotenoid was higher in *I. crithmoides* with a trend toward higher concentrations as the salinity increased. Vitamin B6 displayed similar contents in both species. In *M. nodiflorum*, vitamin B1 tended to increase with salinity, unlike vitamin B6, whose contents remained stable. In *I. crithmoides* the content of these vitamins did not change with the salinity of the cultivation media. In plants, vitamin B1 is considered a growth factor and is distributed between organs, such as leaves, flowers, fruits, seeds and in certain plant’s roots.

Vitamin B1 is a coenzyme in metabolic pathways involved in energy production, carbon assimilation and respiration [38]. It has also been demonstrated that vitamin B1 is an important component in plant stress responses [39]. The values obtained are in accordance with Chamkouri et al. [40], which reported 181 and 102 mg Kg^−1^ dry weight (dw), respectively, in leaves of *Suaeda aegyptiaca* and *Suaeda vera*. These values were also comparable to those found in vegetables naturally rich in this vitamin, such as garlic [34]. Considering that the recommended daily dose (RDD) for this vitamin is 2 mg, the consumption of 20 g of these plants (the estimated dose in a salad or as an ingredient in another dish) will represent 50% and 20% of the DDR for *I. crithmoides* and *M. nodiflorum*, respectively. According to Regulation (EC) No. 1 924/2006 of the European Parliament and of the Council of 20 December 2006 on nutrition and health claims made on foods, a claim that a food is a ‘source of’ vitamins is justified when 100 g (ww) of the product contains at least 15% of the RDD (as defined in the Annex to Directive 90/496/EEC). However, a claim that a food is “rich in” vitamins, is made if it contains at least twice the content required for the “source of” claim. Thus, *I. crithmoides* and *M. nodiflorum* can also be considered plants rich in vitamin B6.

The carotenoid content in *I. crithmoides* increased with increasing salinity in the nutrient solution. The values presented in this study are in accordance with the values obtained in a study conducted in *Inula helenium* L. [41], which were respectively, 19 and 16 µg g^−1^ ww for *β*-carotene and lutein, representing more than 72% of the total carotenoids content. Similar values of *β*-carotene can be found in endives or arugula [34], which are generally considered good sources of vitamin A, since *β*-carotene is metabolized into this vitamin in animal tissues. Lutein is an important pigment for the regeneration and prevention of aging-associated macular degeneration. It is found in foods such as spinach, in relatively high concentrations of 12 mg 100 g^−1^ ww [34]. The analyzed plants have lower concentrations than spinach but are much higher than those found in other halophyte plants, such as *Prosopis strombulifera*, *Sesuvium portulacastrum* or *Tecticornia indica* [42,43].

### 2.5. Antioxidant Activity

Halophyte extracts, including extracts of the *Inula crithmoides* and *Mesembryanthemum nodiflorum* show a moderate phenolic content, abundant interspecific variability for free radical scavenging capacity, and may work as superoxide anion inhibitors [2,44].

In this study, two phenolic groups were evaluated in the acetone extracts of the plants: total phenolic compounds and condensed tannins. The evaluation of total phenolics was performed by testing the reagent Folin-Ciocalteu (F-C reagent), measured as mg GAE (Gallic acid equivalent) per mg of sample, and the condensed tannins assay evaluated the changes caused by salt stress in the content of proanthocyanidins. The results are shown in Table 3.

*I. crithmoides* extracts proved to be susceptible to the loss of the production capacity of phenolic compounds with the increase of the salt stress, presenting a sharp drop at salinities of 100 mmol L^−1^ or higher. This behavior corroborates with Alhdad et al. [45], who stated that the production of phenolics can be induced by several forms of abiotic stress, under which saline stress can promote both the reduction and accumulation of these compounds. Conversely, *M. nodiflorum* extracts showed an increase trend in phenolics up to a salinity of 275 mmol L^−1^, followed by a decrease in these values for higher salinities (350 and 465 mmol L^−1^ of NaCl). Similar responses were observed in other halophyte species, such as *S. ramosissima*, *Atriplex prostrata*, and *Desmostachya bipinnata*, subjected to salt stress [20,46,47]. Such responses can be attributed to the need for resource management by the plants upon exposure to high salt concentrations. Reduced ability to produce phenolic compounds may be a consequence of the need for using the energy available to promote salt exclusion or osmolyte biosynthesis [48]. Care should be taken when considering these results as the F-C reagent can react with other compounds also present in the plants, such as vitamin C and proteins [49,50]. In this study, however, protein content showed an opposite trend to TPC, and such interference may have been minimum.

Previous research has confirmed that phenolic compounds have an important activity as antioxidant agents in halophytes. According to Falleh et al. [44], these antioxidants work as protective agents against stress, which may include the elimination of large amounts of reactive oxygen species (ROS) from cells. This antioxidant capacity can be assessed with the ABTS and DPPH assays that measure the ability of the plant extracts to scavenge free radicals. Usually, a positive relationship is observed between the phenolic compounds content of the extracts and their antioxidant activity. The ABTS assay is recommended for both hydrophilic and lipophilic compounds, while the DPPH method presents better results for compounds soluble in organic solvents [51]. However, both have been widely used due to the high sensitivity in identifying antioxidant compounds at low concentrations.

In Table 3, it is possible to verify that the *I. crithmoides* extracts showed lower capacity to sequester the radical ABTS^+^•, compared to those of *M. nodiflorum*, with values close to or higher than 10 mg/mL (the highest concentration of extract tested). The best result was observed for plants grown at a salinity of 110 mmol L^−1^, resulting in an IC_50_ of 6.12 ± 0.28 mg mL^−1^. However, for *M. nodiflorum* extracts, a continuous significant increase in the antioxidant capacity with salt stress was observed up to a salinity of 275 mmol L^−1^, where the lowest IC_50_ for the ABTS^+^• radical, 2.02 ± 0.05 mg mL^−1^, was obtained (Table 3). Although low, when compared with the positive control (BHT; IC_50_ 0.07 mg mL^−1^), all extracts had the ability to sequester the DPPH radical. Both species showed increased antioxidant capacity with higher salinities, observable from 100 and 275 mmol L^−1^ upward for *I. crithmoides* and *M. nodiflorum* extracts, respectively, similar to what was observed for the ABTS^+^• radical (Table 3). Contrary to what was reported by Doudach et al. [52], who reported a positive relationship between tannins content and other phytoactive compounds concerning the antioxidant capacity of halophyte extracts, no correlation between phenolics or tannins contents and the antioxidant activity was found in this study. Although it is widely accepted that higher phenolic compounds lead to higher antioxidant capacity, studies have not identified any relationship between the concentration of phenolic extracts and increased ability to scavenge free radicals, such as ABTS^+^• and DPPH• in extracts of other halophytes [10,20]. In this perspective, the presence of other antioxidant compounds in halophyte plants, such as vitamins and carotenoid pigments, can be related to changes in the antioxidant capacity of these extracts [53]. Furthermore, from the nutritional point of view, the presence of bioactive compounds and the possibility of improving the capacity of halophytes to produce these compounds through controlled and sustainable cultivation causes these plants to be good candidates for using them as functional food and in biomedical applications.

### 2.6. Sensory Properties

**Figure 3:**
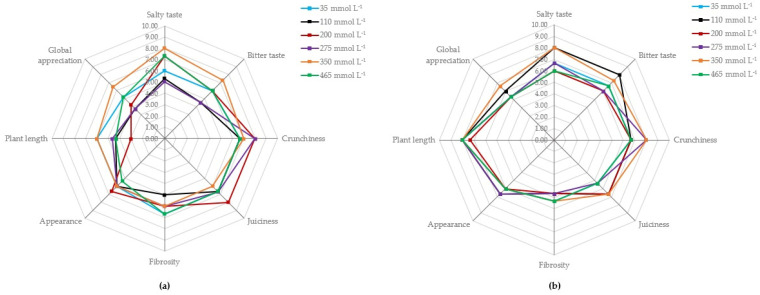
Visual display of the sensory attributes based on the results of the QDA test for *I. crithmoides* (**a**) and *M. nodiflorum* (**b**) cultivated at six different salinities. For each attribute, the ranking increases as it moves farther away from the central point.

The commercial aspect motivated the evaluation of these plants regarding their organoleptic characteristics. For this, a panel of fine-dining chefs as culinary experts, most awarded with Michelin stars, was assembled. The evaluation was based on the QDA test, and the chefs’ panel was responsible for the definition of the sensory attributes to better evaluate the plants, aiming for the product with the best sensory features. Each chef received a set of plants cultivated at different salinities and completed a form with their evaluation of the several parameters, including salty and bitter taste, crunchiness, juiciness, presence of fibers (fibrosity), appearance, and plant length. The answers were ranked, and the results for both species are shown in Figure 3.

Overall, despite a wide range between the lowest and highest cultivation salinity of the samples (35–465 mmol L^−1^), little differences in sensory characteristics were observed. However, for both species, samples grown at 350 mmol L^−1^ (orange line), were better evaluated in the overall assessment.

Halophytes are naturally salty plants and, in this sense, the preference of the panel for a sample saltier than usual can be explained by neuroscience. According to Leshem [54], the human appetite for salt is multifactorial in origin and is expressed habitually and unconsciously, and although we do not prefer eating a spoonful of pure salt, we are usually attracted to salty foods, a will generically defined as “sodium appetite”.

The most obvious effect of reducing NaCl in the culture of the samples was to reduce the salty taste; however, other effects were also observed. Plants grown at 35 mmol L^−1^ had a significantly higher moisture content than samples grown at 465 mmol L^−1^. This may be due to the liquid retention capacity promoted by the increase in NaCl content [55]. Moreover, according to Gaudette and Piestrasik [56], lower NaCl levels can enhance bitterness, decrease positive flavors associated with salinity, and affect the appearance of the food, such as color and texture (significant reduction observed in both species as shown in Table 1). It can also explain why the sample of *I. crithmoides* cultivated at 350 mmol L^−1^ received a better evaluation for the attributes of salty taste, bitter taste, plant length, and global appreciation, whereas *M. nodiflorum* scored better regarding its salty taste, crunchiness, juiciness, fibrosity, and plant length.

Despite the several recent studies with halophyte species for human consumption, few focus their interests on the sensory analysis of these plants. Until now, for the species evaluated in this study cultivated under controlled conditions, we are not aware of any published study on sensory properties, making these results a valuable literary source.

### 2.7. Multivariate Analysis

**Figure 4:**
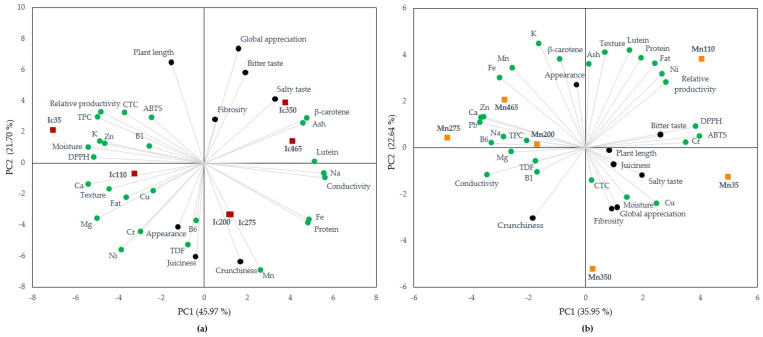
Principal components analysis biplot showing the loadings on PC1 and PC2 of the different parameters of the nutritional profile (green circles), organoleptic characteristics ranking (black circles), and data scores of *I. crithmoides* (**a**) and *M. nodiflorum* (**b**) grown under different salinities (red and orange squares).

To assess the relationships between the different parameters studied and how they were globally influenced by the imposed saline stress, a principal component analysis (PCA) was performed. With PCA, it is possible to visualize the information contained in the various original variables into a smaller set of statistical variables (components) with the minimum loss of information [57]. The analysis was performed separately for each species because the differences between their composition overlapped their response to salinity stress, thus hampering the visualization of such differences. The results are shown in Figure 4.

For *I. crithmoides*, (Figure 4a) the first two principal components explained 67.67% of the total variation of the data (PC1 45.97%; PC2 21.70%), and for *M. nodiflorum* (Figure 4b), explained 58.59% (PC1 35.95%; PC2 22.64%). In the loadings scatter plot of the *I. crithmoides* variables (Figure 4a), the parameters of relative productivity, texture, moisture, fat, TDF, K, Ca, Mg, Cu, Zn, Ni, Cr, Pb, B1, lutein, TPC, CTC, ABTS, and global appreciation were the most discriminant. Certain parameters exhibited significant correlations (*p* < 0.05) with the conductivity of the samples, a parameter that can be used as a proxy for the salinity of the cultivation media. These were: moisture (−0.9487), protein (0.8928), Na (0.9735), K (−0.8753), Ca (−0.9334), Mg (−0.8312), Fe (0.9054), Zn (−0.8149), lutein (0.9163), TPC (−0.9106), and DPPH (−0.9072). For *M. nodiflorum* (Figure 4b), the most discriminant parameters in PC1 were relative productivity, texture, moisture, protein, lipids, Cu, Ni, Cr, lutein, CTC, ABTS, DPPH, salty taste, bitter taste, juiciness, and appearance, several of which also present significant correlations (*p* < 0.05) with conductivity: Na (0.8801), vit B6 (0.8828), and ABTS (−0.8580). Parameters showing positive correlation with conductivity indicate that plants have improved contents with increasing salinities, further indicating that, in this species, these components can be boosted by the application of moderate abiotic stress such as a culture solution with high salinity. However, as lower IC_50_ values for ABTS and DPPH represent higher antioxidant capacity, a negative relationship with conductivity indicates that these defenses may have been induced by salinity stress.

The red and orange squares, respectively in Figure 4a,b, show the data scores of the samples (plants grown at different salinities) for *I. crithmoides* and *M. nodiflorum*. For *I. crithmoides*, their placement along PC1 (the most representative axis) shows a clear distinction between plants cultivated at the lowest salinities (35 and 110 mmol L^−1^) and those cultivated at higher salt concentrations, however for *M. nodiflorum*, this behavior is not as evident. It also shows the better functional profile and organoleptic evaluation of the plants cultivated at salinities ≥ 275 mmol L^−1^, as most of the organoleptic variables (black circles in Figure 4) for both species are positioned in the same quadrant as the 350 mmol L^−1^ data scores, although for *M. nodiflorum* the lowest salinity (35 mmol L^−1^) is also in this quadrant. The enhanced functional profile of *I. crithmoides* grown at higher salinities (Figure 4a), is also evident by the placement of Ic350 and Ic465 data scores in the same quadrant as that of the carotenoid pigments and “global appreciation”, but in the opposite quadrant of the IC_50_ of the DPPH and ABTS assays. For *M. nodiflorum* (Figure 4b), it is apparent the better functional profile of the plants cultivated at 275 and 465 mmol L^−1^ (Mn275 and Mn465), which were positioned in the same quadrant as that of *β*-carotene, and vitamins B1 and B6. Similarly, to *I. crithmoides* these exist in the opposite quadrant in which the IC_50_ of the DPPH and ABTS assays are located.

## 3. Materials and Methods

### 3.1. Plant Material and Growth Conditions

The study was conducted in a polyethylene greenhouse of approximately 200 m^2^ at the facilities of RiaFresh (Portugal) from March to October 2017. I. crithmoides and M. nodiflorum were grown in a closed soilless cultivation system. Seeds were sown directly in substrate and placed in honeycombed trays. Seedlings developed under natural photoperiod conditions and air temperature. The two species studied were distributed over six cultivation tables, each corresponding to a specific salinity treatment. Each table contained six trays, randomly distributed, corresponding to three repetitions for each species in which each repetition occupied an area of approximately 0.18 m^2^.

The nutrient solutions were prepared in tanks of 500 L of capacity using water from a well located nearby the greenhouse. The composition of the solutions is intellectual property of the company and were nutritionally balanced, containing all essential macro- and micronutrients. Additionally, no symptoms of nutritional deficiencies were observed throughout the trial. The solutions were constantly aerated using an air pump and a diffusion system. Plants were irrigated with two daily floods, simulating tides. Six salinity levels were imposed: 35, 110, 200, 275, 350, and 465 mmol L^−1^ NaCl, using NaCl as the salinizing agent. The salinity levels were defined considering the range between a semi-saline medium (<40 mmol L^−1^ NaCl) and the salinity of sea water (630 mmol L^−1^ NaCl). Each salinity level corresponded to an individual tank in which NaCl was added and dissolved in the nutrient solution.

Monitoring of nutrient solutions was conducted through daily measurements of pH (Hanna Instruments) and electrical conductivity (EC, dS m^−1^, Hanna Instruments), whereas nitrate concentration (NO_3_^−^, mg L^−1^) was determined twice a week according to Hoather et al. [58]. Briefly, a sample of the nutrient solution was collected and filtered to remove possible interference from suspended particles and acidified with HCl (1 M), afterward the UV absorption was measured at 220 nm for the determination of NO_3_^−^, and a second measurement at 275 nm was used to correct the NO_3_^−^ value. Absorption measurements were performed using a UV-Visible Spectrophotometer (UV-160 A, Shimadzu Kyoto, Japan). During the experiment, the solutions were refilled twice a week and completely replaced at least once a month.

Harvesting was performed when the stems of each species reached at least 20 cm height. The cuttings were performed using pruning shears and the fresh weight of the collected biomass was determined immediately. The productivity was quantified based on the fresh biomass of the aerial part harvested by area and time after sowing (g m^−2^ day^−1^). The relative productivity was calculated for each salinity level and expressed as a percentage of variation compared to the lower salinity (35 mmol L^−1^). A sample of the aboveground biomass from each treatment and from each repetition was collected and transported to the laboratory in a refrigerated bag. Samples were frozen (−20 °C) and later freeze-dried and milled in a planetary ball mill (Retsch-PM 100, Retsch GmbH, Haan, Germany). Powdered samples were kept in a desiccator until the analyses.

### 3.2. Nutritional Characterization

#### 3.2.1. Proximal Composition

The fresh biomass was used to determine the moisture content in a forced air circulation oven at 105 °C for 16 h. To translate the salty flavor of the fresh plants, the samples were macerated with distilled water at a ratio of 1:10 (*w*/*w*) and the electric conductivity (EC) measured using a conductivity meter (Hanna Instruments).

The remaining parameters were determined on freeze-dried biomass for 48 h at −55 °C (LyoAlfa 10/15-Telstar, Spain). Total protein content was estimated by the total N content (conversion factor: 6.25) measured in an Elemental Analyzer model Vario III (Vario EL, Germany). The ash content was determined by incineration at 550 ± 15 °C for 6 h in a muffle furnace (Nabertherm GmbH, L 3/12). Crude fat was determined with the conventional single-phase extraction method [59] with some modifications; briefly, dried biomass was homogenized in chloroform, methanol, and water (2:2:1, *w*/*v*/*v*) in an Ultra-Turrax disperser (IKA-Werke GmbH Staufen, Germany) and the lipidic phase was collected and evaporated in a dry bath. The result was obtained by weight difference. Total dietary fiber (TDF) was determined according to McCleary et al. [60] by an enzymatic-gravimetric method, as described in the kit manual provided by Megazyme (Megazyme International Ireland Limited, Bray, Ireland), based on the AACC method 32–05.01 and AOAC method 985.29, and expressed as g 100 g^−1^ ww.

#### 3.2.2. Firmness

The firmness was determined as resistance to penetration measured with a texturometer (LFRA Texture Analyzer, Brookfield) equipped with a 1.5 kg cell. According to the description by Taniwaki et al. [61], each sample was placed between two metal plates with a cylindrical hole in the center (Ø = 15 mm) that penetrated until the samples broke. A stainless-steel drilling probe (TA-39) with a diameter of 2 mm and a length of 20 mm was used. For each sample, five replicates were performed, and the probe penetration force was measured in grams (g).

### 3.3. Minerals

In triplicate, the ashes of each species were digested with a mixture of 65% nitric acid (HNO_3_) and hydrogen peroxide (H_2_O_2_) 2:1 (*v*:*v*) under constant heating until a white homogeneous precipitate was obtained, followed by dilution of the sample in 5% HNO_3_. Mineral elements were analyzed using a microwave plasma-atomic emission spectrometer (MP-AES; Agilent 4200 MP-AES, Australia). The blanks values were subtracted to correct the final concentrations of analyzed metals. Results were expressed as mg or μg g^−1^ wet weight (ww).

### 3.4. Vitamins and Carotenoids

The extraction of water-soluble vitamins and carotenoids was based on the modified version of Santos et al. [62] and the respective contents were quantified by HPLC-DAD using the method proposed by Klejdus et al. [63]. The procedure was described in detail in a previous study [20]. In brief, 0.25 g of freeze-dried powder were extracted with ammonium acetate (10 mmol L^−1^) and a methanol solution (50:50 *v*/*v*) containing 0.01% of butylated hydroxytoluene (BHT) to prevent oxidation. Hippuric acid (2.5 µg mL^−1^) was added as an internal standard. Samples were homogenized and sonicated in an ultrasound bath and then centrifuged at 12,500 g for 20 min. The supernatant containing the water-soluble vitamins was placed under a gentle nitrogen stream to evaporate the methanol, the final volume was registered, and the samples were filtered through a 0.22 µm nylon filter membrane and stored at −80 °C until analysis. For the carotenoid content, the pellet was extracted twice with ethyl acetate, the supernatants combined, dried, resuspended in 1mL of HPLC grade ethyl acetate, filtered through a 0.22 µm PTFE filter, and stored at −80 °C until analysis.

Quantification was performed in a Dionex HPLC Analytical System (Sunnyvale, CA, USA) equipped with a photodiode array detector and fitted with an automated sample injector. The chromatographic separation was achieved using a Purospher^®^ STAR RP-18 endcapped (Merck) (250 × 2.1 mm, 5 µm) column. For the water-soluble vitamins, the column was stabilized at 25 °C and a 10 µL aliquot was injected. A gradient mobile phase at a flow rate of 0.6 mL min^−1^ composed of acetonitrile as solvent A and 0.01% trifluoroacetic acid (TFA) as solvent B was used. The gradient program applied was the following: 0–3 min 8% A; 3–11 min 8–98% A; 11–30 min 98% A. For the carotenoids, the column was stabilized at 20 °C and a 100 µL aliquot was injected. A gradient mobile phase at a flow rate of 1 mL min^−1^ composed of 9:1 (*v*/*v*) acetonitrile and water as solvent A and ethyl acetate as solvent B. The gradient program was: 0–16 min from 100% to 40% A; hold for 14 min; 100% B in 2 min; hold for 3 min; 100% A in 5 min. The water-soluble vitamins were quantified at a 280 nm wavelength and the carotenoids at 450 nm. A spectrum between 180 nm and 800 nm was recorded to assess peak purity and confirm the identity of the compounds.

Thiamine (B1), pyridoxine (B6), lutein and *β*-carotene were obtained from Sigma Aldrich and were of standard quality. Stock standard solutions were prepared in either Milli-Q water or methanol (HPLC grade) and standard curves were prepared for each compound. Identification was performed using the retention times compared to those of the pure standard solutions. Quantification was conducted using the peak areas and the final values were expressed in µg (or mg) per 100 g of biomass (ww).

### 3.5. Antioxidant Capacity

For the analyses of antioxidant activity, extracts were prepared from the dried biomass powder with 80% acetone, as described by Lima et al. [20].

#### 3.5.1. Radical Scavenging Activity (RSA)

RSA on ABTS radical was evaluated following the method described by Re et al. [64]. Briefly, in 96-well microplates, the ABTS radical was prepared fresh until an absorbance of approximately 0.7 at 734 nm was obtained. At a later stage, 10 μL of the extract was mixed with 190 μL of the ABTS solution. The mixture was incubated for 6 min in the dark at room temperature (RT) and the absorbance was measured at 734 nm. A solution containing 10 μL of extract and ethanol was used as the color control.

RSA on the DPPH radical was evaluated by the method described by Moreno et al. [65]. In 96-well plates, 22 μL of the extract was mixed with 200 μL of DPPH 120 μM, previously prepared in methanol. The mixture was incubated at RT for 30 min, and the absorbance was measured at 517 nm. To eliminate any interference from the color of the extracts, a color control was created by mixing 22 μL of extract and methanol. In both methods, a positive control with BHT was performed and activity was expressed as percentage relative to a negative control containing DMSO instead of sample. Moreover, for the extracts displaying activity greater than 50% (at a concentration of 10 mg mL^−1^), the mean inhibitory concentrations (IC_50_) were determined.

#### 3.5.2. Total Phenolics Content (TPC) and Condensed Tannins Content (CTC)

The total phenolics (TPC) and condensed tannins contents (CTC) were determined by spectrophotometry. TPC was determined by the Folin-Ciocalteu assay, with modifications [66]. Absorbance was measured at 725 nm and gallic acid was used as a standard. Results were expressed in gallic acid equivalents per gram of dried extract (mg GAE g^−1^ dw). Tannins content (CTC) was evaluated by the colorimetric method of 4-dimethylaminocinnamaldehyde hydrochloric acid (DMACA-HCl) [67], with the adaptations created by Zou et al. [68] for 96-well microplates. Absorbance was measured at 640 nm and the results were expressed in milligrams of catechin equivalents per gram of dried extract (mg CE g^−1^ dw). All assays were performed in a microplate reader (Biotek Synergy 4, Biotek Instruments, Winooski, VT, USA).

#### 3.5.3. Radical Scavenging Activity (RSA)

RSA on ABTS radical was evaluated following the method described by Re et al. [64]. Briefly, in 96-well microplates, the ABTS radical was prepared fresh until an absorbance of approximately 0.7 to 734 nm was obtained. At a later stage, 10 μL of the extract was mixed with 190 μL of the ABTS solution. The mixture was incubated for 6 min in the dark at RT and the absorbance was measured at 734 nm. A solution containing 10 μL of extract and ethanol was used as the color control.

RSA on DPPH radical was evaluated by the method described by Moreno et al. [65]. Using 96-well plates, 22 μL of the extract was mixed with 200 μL of DPPH 120 μM, previously diluted in methanol. The mixture was incubated at RT for 30 min, and the absorbance was measured at 517 nm. To eliminate any interference from the color of the extracts, a color control was made by mixing 22 μL of extract and methanol. In both methods, positive control with BHT was performed and activity was expressed as a percentage relative to a negative control containing DMSO instead of sample. Moreover, for the extracts displaying activity greater than 50% (at a concentration of 10 mg mL^−1^), the mean inhibitory concentrations (IC_50_) were determined.

#### 3.5.4. Total Phenolic Content (TPC) and Condensed Tannin Content (CTC)

Spectrophotometry was the method used to evaluate the total phenolic content (TPC) and condensed tannin content (CTC). TPC was determined by the Folin-Ciocalteu assay [66], with modifications. Absorbance was measured at 725 nm and gallic acid was used as a standard. Results were expressed in gallic acid equivalents per gram of dried extract (mg GAE g^−1^ dw). Tannin content (CTC) was evaluated by the colorimetric method of 4-dimethylaminocinnamaldehyde hydrochloric acid (DMACA-HCl) [67], with adaptations created by Zou et al. [68] for 96-well microplates. Absorbance was measured at 640 nm and the results were expressed in milligrams of catechin equivalents per gram of dried extract (mg CE g^−1^ dw). All assays were performed in a microplate reader (Biotek Synergy 4, Biotek Instruments, Winooski, VT, USA).

### 3.6. Sensory Analytical Method

The organoleptic characteristics of the halophyte plants grown in different salinities were evaluated using the quantitative descriptive analysis (QDA) as described by Stone et al. [69], with slight modifications as the place where the tests were performed and the manner the answers were obtained. The experiment started with the choice of attributes, in this case, attributes for flavor (salty and bitter taste), consistency or texture felt in the mouth (crunchiness, juiciness, and fibrosity), and physical (appearance, plant length) characteristics, as well as global appreciation. The intensity of each attribute was measured on an effective scale of four options; for salty and bitter taste, crunchiness, and juiciness characteristics from “not enough” to “too much”; for fibrosity, from “excessive” to “non-existent”; for plant length, from “too short” to “too long”; and for appearance and global appreciation, from “very bad” to “excellent”.

The panel of experts was composed of 10 awarded fine-dining chefs, all with previous knowledge on the use of halophyte plants in gourmet cuisine, and highly experienced in sensory evaluation, fulfilling the requirements of the QDA methodology [70]. All experts were contacted previously to explain the objectives of the sensory evaluation and to engage their commitment. The formulation of the questionnaires was created together with one of the experts to ensure that the language and terminology was clear and accurate. Thus, considering the experience and commitment of the judges, the training and validation stages were considered unnecessary. The samples were therefore shipped to the chefs and the evaluation performed in their respective work environments and answers completed online or sent by mail.

### 3.7. Statistical Analysis

All experiments were performed at least in triplicate and results were expressed as mean ± standard error of the mean (SEM). Statistical analysis was performed using Statistica 7.0 software (Statsoft Inc., USA), and significant differences were assessed by analysis of variance (ANOVA) using the Tukey HSD (honestly significant differences) test, or the Duncan’s new multiple range test when parameterization of the data did not prevail. The IC_50_ values were calculated by the sigmoidal fitting of the data with the GraphPad Prism v.7.0 software.

Principal components analysis (PCA) was performed for the global data set to assess the interrelationships between variables and obtain a better perception of the different parameters studied using the XLSTAT statistical add-on for excel (Addinsoft Inc, New York, NJ, USA).

## 4. Conclusions

*Inula crithmoides* and *Mesembryanthemum nodiflorum* are halophytes that present good adaptation to soilless cultivation systems, a sustainable technique able to produce nutritionally interesting plants, promoting the improvement of essential nutrients for human consumption, such as proteins, minerals, total dietary fiber, and low lipid content. The salinity of the cultivating media significantly improved the nutritional profile, especially the antioxidant capacity of the plants and their carotenoid and vitamins contents, but negatively influenced biomass productivity. The salinity of cultivation media was also important for defining the preferred samples in the sensory test, with emphasis on samples grown at 350 mmol L^−1^ for both species.

## Figures and Tables

**Table 1:** Relative productivity, electric conductivity, texture, and proximate composition of the samples cultivated at six different salinities. Values are presented as means ± SEM.

Salinity (mmol L^−1^)	35	110	200	275	350	465
Relative productivity (%)						
*I. crithmoides*	100 ± 10 ^a^	42.9 ± 2.7 ^bc^	29.2 ± 3.3 ^c^	33.1 ± 2.2 ^bc^	36.5 ± 3.5 ^bc^	33.3 ± 3.9 ^bc^
*M. nodiflorum*	100 ± 1 ^a^	102 ± 9 ^a^	67 ± 19.6 ^ab^	67.5 ± 10.9 ^ab^	42.2 ± 5.9 ^bc^	58.2 ± 3.2 ^bc^
Conductivity (dS m^−1^)						
*I. crithmoides*	3.15 ± 0.14 ^f^	4.17 ± 0.03 ^e^	5.29 ± 0.09 ^d^	5.17 ± 0.09 ^d^	5.52 ± 0.02 ^d^	5.73 ± 0.37 ^cd^
*M. nodiflorum*	5.76 ± 0.24 ^cd^	6.40 ± 0.09 ^c^	7.40 ± 0.19 ^b^	8.17 ± 0.05 ^a^	8.37 ± 0.28 ^a^	8.60 ± 0.08 ^a^
Firmness (g)						
*I. crithmoides*	181 ± 19 ^cd^	170 ± 17 ^cdef^	174 ± 20 ^cde^	148 ± 15 ^ef^	144 ± 14 ^f^	154 ± 10 ^def^
*M. nodiflorum*	258 ± 16 ^b^	301 ± 27 ^a^	258 ± 19 ^b^	248 ± 26 ^b^	na	186 ± 32 ^c^
Moisture (%)						
*I. crithmoides*	88.1 ± 0.6 ^ab^	86.6 ± 0.5 ^bc^	86.1 ± 0.4 ^c^	85.1 ± 0.2 ^c^	85.3 ± 0.4 ^c^	85.0 ± 0.6 ^c^
*M. nodiflorum*	89.3 ± 0.3 ^a^	88.1 ± 0.1 ^ab^	89.8 ± 0.9 ^a^	88.0 ± 0.5 ^ab^	88.6 ± 0.3 ^a^	86.8 ± 0.6 ^bc^
Ash (g 100 g^−1^ ww)						
*I. crithmoides*	4.26 ± 0.15 ^de^	4.95 ± 0.16 ^bc^	4.61 ± 0.13 ^cd^	4.94 ± 0.01 ^bc^	5.36 ± 0.05 ^ab^	5.70 ± 0.06 ^a^
*M. nodiflorum*	4.09 ± 0.18 ^de^	4.65 ± 0.15 ^cd^	4.06 ± 0.09 ^de^	3.95 ± 0.46 ^e^	3.89 ± 0.06 ^e^	5.04 ± 0.12 ^bc^
Protein (g 100 g^−1^ ww)						
*I. crithmoides*	2.76 ± 0.15 ^b^	3.01 ± 0.08 ^b^	3.18 ± 0.34 ^b^	3.29 ± 0.33 ^ab^	3.13 ± 0.09 ^b^	3.17 ± 0.03 ^a^
*M. nodiflorum*	1.55 ± 0.13 ^c^	1.95 ± 0.02 ^c^	1.49 ± 0.24 ^c^	1.40 ± 0.16 ^c^	1.42 ± 0.06 ^c^	1.71 ± 0.14 ^c^
Fat (g 100 g^−1^ ww)						
*I. crithmoides*	0.59 ± 0.09 ^bc^	0.91 ± 0.06 ^a^	0.57 ± 0.08 ^bc^	0.49 ± 0.06 ^bcd^	0.43 ± 0.02 ^cd^	0.41 ± 0.03 ^cd^
*M. nodiflorum*	0.55 ± 0.06 ^bcd^	0.67 ± 0.07 ^b^	0.48 ± 0.02 ^cd^	0.47 ± 0.02 ^d^	0.36 ± 0.02 ^cd^	0.44 ± 0.02 ^d^
TDF (g 100 g^−1^ ww)						
*I. crithmoides*	1.85 ± 0.01 ^c^	1.93 ± 0.01 ^b^	1.84 ± 0.01 ^d^	2.07 ± 0.01 ^a^	1.82 ± 0.01 ^e^	1.81 ± 0.01 ^f^
*M. nodiflorum*	1.35 ± 0.01 ^k^	1.45 ± 0.01 ^j^	1.18 ± 0.01 ^l^	1.74 ± 0.01 ^g^	1.58 ± 0.01 ^h^	1.52 ± 0.01 ^i^

na: not available; ww: wet weight; TDF: total dietary fiber; In each analysis (two lines), different letters indicate significant differences between different salinities (*p* < 0.05).

**Table 2:** Mineral composition of *I. crithmoides* and *M. nodiflorum* cultivated at six different salinities.

Salinity (mmol L^−1^)	35	110	200	275	350	465
*Inula crithmoides*						
Na (mg g^−1^)	7.01 ± 0.7 ^d^	8.95 ± 0.2 ^c^	12.0 ± 0.1 ^b^	13.5 ± 0.4 ^a^	13.7 ± 0.1 ^a^	14.4 ± 0.2 ^a^
K (mg g^−1^)	3.10 ± 0.33 ^a^	3.13 ± 0.02 ^ab^	2.55 ± 0.04 ^bc^	2.78 ± 0.13 ^c^	2.67 ± 0.01 ^c^	2.68 ± 0.08 ^c^
Ca (mg g^−1^)	1.58 ± 0.13 ^a^	1.46 ± 0.01 ^ab^	1.33 ± 0.01 ^b^	1.41 ± 0.10 ^ab^	1.22 ± 0.10 ^b^	1.28 ± 0.01 ^b^
Mg (mg g^−1^)	0.96 ± 0.08 ^a^	0.96 ± 0.01 ^ab^	0.86 ± 0.01 ^ab^	0.90 ± 0.09 ^ab^	0.74 ± 0.14 ^ab^	0.75 ± 0.04 ^b^
Fe (µg g^−1^)	8.78 ± 1.05 ^c^	10.5 ± 0.15 ^bc^	11.7 ± 0.13 ^ab^	11.8 ± 0.84 ^a^	10.8 ± 0.29 ^ab^	12.3 ± 0.12 ^a^
Cu (µg g^−1^)	0.87 ± 0.13 ^a^	0.63 ± 0.03 ^bc^	0.74 ± 0.06 ^abc^	0.77 ± 0.03 ^ab^	0.57 ± 0.05 ^c^	0.79 ± 0.02 ^ab^
Mn (µg g^−1^)	5.51 ± 0.48 ^c^	6.00 ± 0.06 ^bc^	7.01 ± 0.03 ^a^	6.82 ± 0.04 ^a^	5.61 ± 0.01 ^c^	6.54 ± 0.07 ^ab^
Zn (µg g^−1^)	2.51 ± 0.14 ^a^	2.70 ± 0.29 ^a^	1.40 ± 0.15 ^bc^	1.56 ± 0.17 ^bc^	1.36 ± 0.04 ^c^	1.86 ± 0.02 ^b^
Ni (µg g^−1^)	0.22 ± 0.05 ^a^	0.23 ± 0.03 ^a^	0.26 ± 0.05 ^a^	0.18 ± 0.01 ^b^	nd	nd
Cr (µg g^−1^)	0.07 ± 0.02 ^a^	0.05 ± 0.01 ^a^	0.06 ± 0.01 ^a^	0.06 ± 0.01 ^a^	nd	0.05 ± 0.01 ^a^
*Mesembryanthemum nodiflorum*						
Na (mg g^−1^)	6.46 ± 2.13 ^b^	7.92 ± 3.30 ^ab^	8.67 ± 1.70 ^ab^	9.81 ± 0.23 ^ab^	9.95 ± 1.80 ^ab^	13.4 ± 1.3 ^a^
K (mg g^−1^)	1.42 ± 0.12 ^c^	2.93 ± 1.70 ^a^	1.93 ± 0.9 ^ab^	2.03 ± 0.17 ^b^	0.97 ± 0.07 ^c^	2.00 ± 0.46 ^ab^
Ca (mg g^−1^)	0.16 ± 0.01 ^a^	0.27 ± 0.25 ^a^	0.34 ± 0.04 ^a^	0.41 ± 0.12 ^a^	0.27 ± 0.24 ^a^	0.33 ± 0.4 ^a^
Mg (mg g^−1^)	0.15 ± 0.02 ^c^	0.14 ± 0.01 ^c^	0.24 ± 0.02 ^a^	0.20 ± 0.01 ^b^	0.16 ± 0.01 ^c^	0.16 ± 0.01 ^c^
Fe (µg g^−1^)	1.08 ± 0.03 ^b^	1.44 ± 0.50 ^ab^	1.82 ± 0.11 ^ab^	1.75 ± 0.22 ^ab^	1.10 ± 0.26 ^b^	1.96 ± 0.32 ^a^
Cu (µg g^−1^)	1.89 ± 0.03 ^a^	0.88 ± 0.02 ^c^	0.94 ± 0.01 ^c^	0.85 ± 0.04 ^c^	1.25 ±0.20 ^b^	1.03 ± 0.12 ^bc^
Mn (µg g^−1^)	5.30 ± 0.52 ^c^	7.50 ± 1.19 ^b^	8.90 ± 0.41 ^ab^	8.12 ± 0.42 ^b^	5.19 ± 0.34 ^c^	10.1 ± 1.1 ^a^
Zn (µg g^−1^)	5.61 ± 0.02 ^f^	8.87 ± 0.37 ^e^	19.4 ± 0.1 ^c^	58.4 ± 0.1 ^a^	10.8 ± 0.3 ^d^	43.0 ± 1.1 ^b^
Ni (µg g^−1^)	0.31 ± 0.02 ^ab^	0.37 ± 0.04 ^a^	0.15 ± 0.01 ^c^	0.16 ± 0.1 ^c^	0.15 ± 0.05 ^c^	0.29 ± 0.03 ^b^
Cr (µg g^−1^)	1.63 ± 0.53 ^a^	1.06 ± 0.36 ^ab^	0.13 ± 0.04 ^c^	0.17 ± 0.02 ^c^	0.60 ± 0.22 ^bc^	0.69 ± 0.25 ^bc^
Pb (µg g^−1^)	0.12 ± 0.01 ^b^	0.11 ± 0.01 ^b^	0.20 ± 0.02 ^a^	0.21 ± 0.02 ^a^	0.12 ± 0.01 ^b^	0.20 ± 0.01 ^a^
Na:K ratio						
*I. crithmoides*	2.26	2.85	4.70	4.85	5.13	5.37
*M. nodiflorum*	4.54	2.70	4.49	4.83	10.2	6.7

Limits of detection (LOD): Cr-0.01 µg L^−1^, Ni-0.02 µg L^−1^, Pb-0.01 µg L^−1^; In each row, different letters indicate significant differences (*p* < 0.05).

**Table 3:** Total phenolics contents (TPC; mg GAE g^−1^ dw), condensed tannin content (CTC; mg CE g^−1^ dw), and antioxidant activity-radical scavenging (IC_50_ mg mL^−1^ of extract) of *I. crithmoides* and *M. nodiflorum* cultivated at six different salinities.

Salinity (mmol L^−1^)	35	110	200	275	350	465
*Inula crithmoides*						
TPC	16.5 ± 3.3 ^a^	10.5 ± 1.9 ^b^	7.04 ± 0.50 ^b^	7.85 ± 0.84 ^b^	7.39 ± 0.90 ^b^	9.06 ± 0.77 ^b^
CTC	42.5 ± 1.8 ^ab^	45.8 ± 2.5 ^a^	28.6 ± 2.0 ^d^	33.5 ± 2.6 ^cd^	34.8 ± 0.8 ^c^	37.2 ± 0.5 ^bc^
Radical Scavenging Activity						
ABTS	9.20 ± 0.09 ^a^	6.12 ± 0.28 ^b^	8.18 ± 0.98 ^a^	>10	9.10 ± 0.59 ^a^	>10
DPPH	5.04 ± 0.43 ^a^	3.89 ± 0.37 ^b^	3.96 ± 0.29 ^b^	3.88 ± 0.13 ^b^	3.59 ± 0.27 ^b^	3.40 ± 0.48 ^b^
*Mesembryanthemum nodiflorum*						
TPC	2.90 ± 0.77 ^bc^	2.24 ± 1.15 ^c^	3.77 ± 1.73 ^ab^	4.39 ± 1.35 ^a^	2.09 ± 0.2 ^c^	2.16 ± 0.12 ^c^
CTC	18.6 ± 1.1 ^b^	17.3 ± 0.6 ^c^	22.6 ± 0.8 ^a^	18.0 ± 0.7 ^bc^	18.1 ± 1.2 ^bc^	14.2 ± 0.6 ^d^
Radical Scavenging Activity						
ABTS	6.65 ± 0.01 ^a^	6.58 ± 0.01 ^a^	3.46 ± 0.16 ^c^	2.02 ± 0.05 ^d^	4.19 ± 0.31 ^b^	3.18 ± 0.02 ^c^
DPPH	4.56 ± 0.50 ^a^	4.81 ± 1.74 ^a^	3.73 ± 0.53 ^ab^	2.75 ± 0.58 ^b^	3.77 ± 0.83 ^ab^	3.54 ± 0.09 ^ab^

ABTS-2,2′-azino-bis(3-ethylbenzothiazoline-6-sulfonic acid); DPPH-1,1-diphenyl-2-picrylhydrazyl radical. In each row, different letters represent significant differences (*p* < 0.05). The IC_50_ (mg mL^−1^) of positive controls were as follows: ABTS (BHT-0.14); DPPH (BHT-0.11).

## Sample Availability

Samples of the cultivated plants can be supplied by RiaFresh.
